# Nasopharyngeal Carcinoma With Skull Base Erosion Cytologic Findings

**Published:** 2012-08-30

**Authors:** Negar Azarpira, Musa Taghipour, Masumeh Pourjebely

**Affiliations:** 1 Department of Pathology, Shiraz University of Medical Sciences, Shiraz, IR Iran; 2 Department of Neurosurgery, Shiraz University of Medical Sciences, Shiraz, IR Iran

**Keywords:** Nasopharyngeal carcinoma, Cytology, Imprinting (Psychology), Intraoperative Period

## Abstract

Nasopharyngeal carcinoma (NPC) occurs more frequently in patients with south-east Asian racial backgrounds. This disease may spreads superiorly to the skull base and intracranium followed by skull base destruction. We report a 56 year-old man presented with headache and diplopia. Magnetic resonance imaging (MRI) revealed extension of destructive mass from ethmoid sinus to the parenchyma. Intraoperative touch cytology showed loose syncytial sheets of pleomorphic abnormal epithelial cells, dyskeratotic cells with abnormal chromatin clumping and irregular nuclear outlines, in a necrotic background. These findings were infavor of keratizing squamous cell carcinoma which was confirmed by histopathology. During interpretation of intraoperative imprint cytology of central nervous system tumors, the possibility of local invasive tumors like NPC should be considered.

## 1. Introduction

NPC is a rare tumor in many parts of the world, but is prevalent among Southeast Asian. It usually arises from eustachian tube opening in the Rosenmuller fossa. Initial complaints are often due to middle ear obstruction (otitis media or hearing loss) or local invasion (headache and cranial nerve palsy). There are few reports of NPC with diffuse skull base destruction, resulting in obstructive hydrocephalus, cerebellopontine involvement or even cavernous sinus metastasis [1][2][3][4]. According to WHO classification, this entity is classified as three different entities: keratizing squamous cell carcinoma, non-keratinizing carcinoma and undifferentiated carcinoma (lymphoepithelioma). Keratizing squamous cell carcinoma mainly developed in male adult patients is least radiosensitive and poor prognosis. Genetic factors and EBV infection have been associated with undifferentiated carcinoma [5].

## 2. Case Report

**Figure 1:**
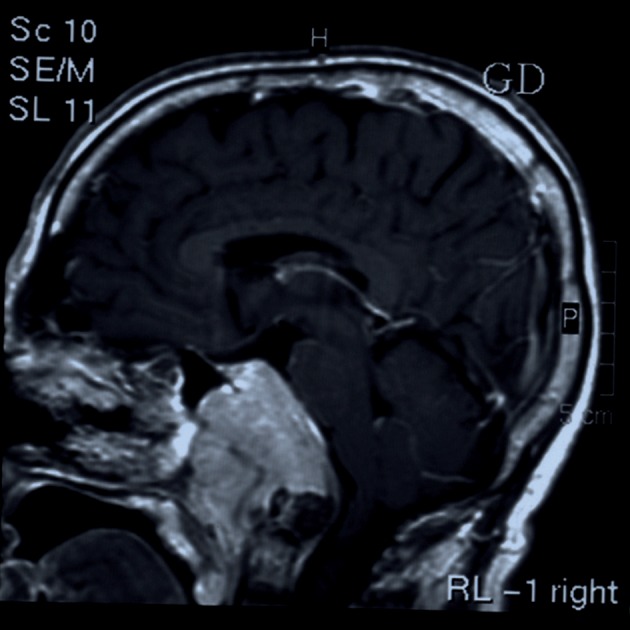
Sagital view in MRI; Nasopharyngeal mass with enhancement after gadolinium injection.

We report a 56 year-old man presented with headache, dizziness and diplopia for one-month duration. On physical examination third and sixth cranial nerve palsy were detected. Past medical history was not significant. MRI showed a soft tissue mass originates from ethmoid sinus with upward extension to brain parenchyma (Figure 1) [6]. The patient underwent surgery with resection of the lesion in the neurosurgery department. Touch preparation and cryosection slides were fixed in alcohol and stained with rapid hematoxylin and eosin (H & E). The cellular smear composed of loose syncytial sheets and clusters of pleomorphic epitheloid cells containing coarse condensated chromatin, irregular nuclear contours, inconspicuous nucleoli with scant keratinized cytoplasm (Figure 2a & 2b). Cytological diagnosis was infavor of keratizing squamous cell carcinoma [5][7]. Histological examination of the resected specimen confirmed the diagnosis (Figure 3).

**Figure 2a:**
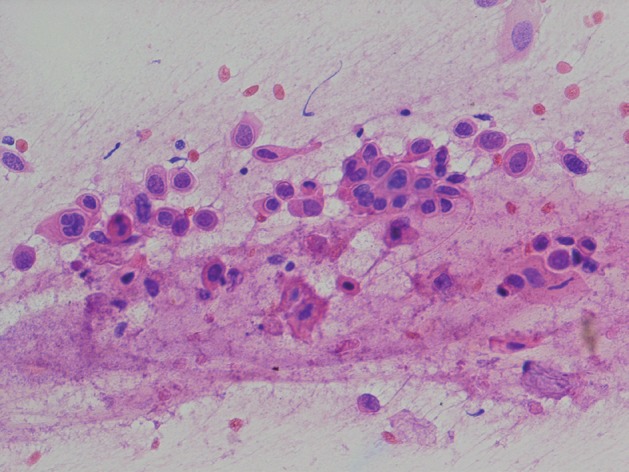
Sagital view in MRI; Nasopharyngeal mass with enhancement after gadolinium injection.

**Figure 2b:**
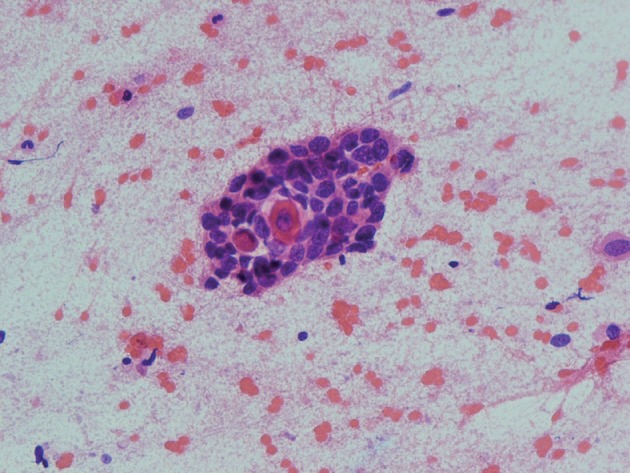
Small single keratized cell in the cluster of atypical cells with coarse condensated chromatin. (H & E ×400)

**Figure 3:**
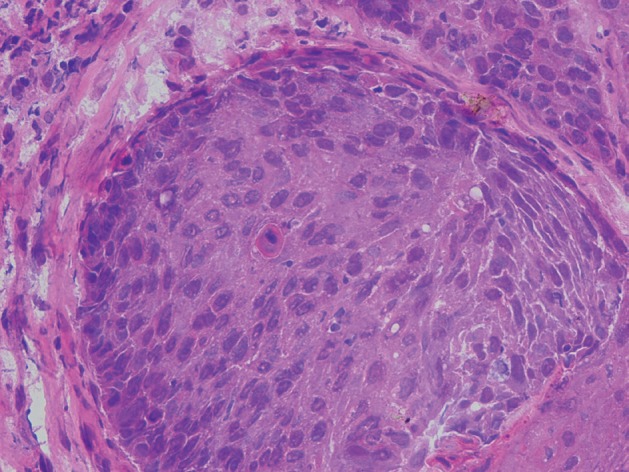
Squamous cell carcinoma with focal evidence of individual cell keratinization. (H & E ×400)

## 3. Discussion

NPC is endemic in Southern China and is associated with Epstein–Barr virus (EBV) as strong aetiological factor. The titre levels of antibodies to EBV immunoglobulin A viral capsid antigen (IgA VCA) and early antigen (IgA EA) have been widely used as screening and diagnostic markers for NPC. Quantitation of EBV DNA viral load using a real-time PCR technique is highly sensitive and specific method for NPC and correlates well with tumor burden. EBV DNA can be used clinically to monitor disease response and recurrence [8][9]. NPC can spread superiorly to the skull base. Cytologically, the differential diagnoses include craniopharyngioma, epidermoid /dermoid cysts Rathke’s cleft cyst. However, the smears of these lesions show sheets of benign transitional or squamoid epithelial cells with a palisade borders as well as wet keratin. There is no evidence of abnormal dyskeratotic cells. These lesions are often cystic and cholesterol crystals or keratin material can often be detected [5][7][10]. On the other hand, NPC particularly nonkeratinizing undifferentiated form, may mimic inflammatory and lymphoproliferative disorders. However, the cohesive nature of the tumor cells can differentiate NPCs from most lymphomas [5][7].

In our experience, imprint cytology test is a rapid and inexpensive diagnostic test that tissue integrity is well preserved. In interpretation of imprint cytology of central nervous system tumors, the possibility of local invasive tumors such as NPC should be considered. In these cases, careful examination of the nasopharynx is essential for the exclusion of nasopharyngeal undifferentiated carcinoma because of the obvious differences in treatment and prognosis.
